# A novel NOTCH3 mutation and its clinical, neuroimaging and pathological presentation in a Chinese patient with CADASIL: A case report

**DOI:** 10.1097/MD.0000000000028870

**Published:** 2022-02-18

**Authors:** Jing Dang, Shengsuo Lei, Mingwan Xia, Jihua Chen

**Affiliations:** Department of Neurology, Chenzhou No.1 People's Hospital, Chenzhou, Hunan, China.

**Keywords:** cerebral autosomal dominant arteriopathy with subcortical infarcts and leucoencephalopathy, China, granular osmiophilic materials, *NOTCH3* gene, novel mutation

## Abstract

**Rationale:**

Cerebral autosomal dominant arteriopathy with subcortical infarcts and leukoencephalopathy (CADASIL) is the most common form of familial cerebral small vessel disease in adults, and is caused by NOTCH3 mutations. However, individual symptom types, onset, and disease severity span a wide range.

**Patient concerns:**

Herein, we report a case of chronic neurological symptoms including slurring of speech, recurrent weakness in both limbs and legs, and progressive memory loss. Cranial magnetic resonance imaging revealed recurrent acute lacunar subcortical infarction and extensive white matter hyperintensities. Skin biopsy revealed granular osmiophilic materials close to the cell surface of smooth muscle cells in an arteriolar vessel. The patient's genomic DNA showed a mutation c.635G>C[p.(Cys212Ser)] in exon 4.

**Diagnosis:**

The patient was finally diagnosed with CADASIL.

**Interventions:**

The patient was treated with antiplatelet therapy and extremity rehabilitation.

**Outcomes:**

There was no improvement in speech, extremity function, or memory.

**Lessons:**

Accurate early diagnosis and appropriate treatment are crucial to improve the prognosis of patients with CADASIL.

## 1 Introduction

CADASIL is the most prevalent inherited cause of cerebral small-vessel disease in adult.^[1]^ Its characteristic clinical manifestations include migraine with or without aura, recurrent transient ischemic attacks and stroke in subcortical regions, psychiatric disorders, and cognitive decline.^[2]^ Brain magnetic resonance imaging (MRI) reveals diffuse white matter hyperintensities, especially in the anterior-temporal lobe and external capsule, and multiple lacunar infarcts.^[3]^ The pathological hallmark of CADASIL is the presence of granular osmiophilic material (GOM) in the basement membrane of vascular smooth muscle cells. This disease is caused by mutations in NOTCH3, which encodes epidermal growth factor-like repeats (EGFRs). Most NOTCH3 mutations reported to date have resulted in missense mutations that lead to the loss of cysteine residues.^[4]^ More than 200 different NOTCH3 mutations have been reported worldwide (HGMD website; http://www.hgmd.cf.ac.uk/). There is almost no genotype-phenotype correlation between certain NOTCH3 mutations and CADASIL symptoms.^[4]^ The extremely variable phenotype makes the clinical diagnosis of CADASIL challenging.^[5]^

Here, we report the clinical, neuroimaging, and pathological features of a newly diagnosed patient with CADASIL with a novel NOTCH3 mutation.

## 2 Case report

**Figure 1:**
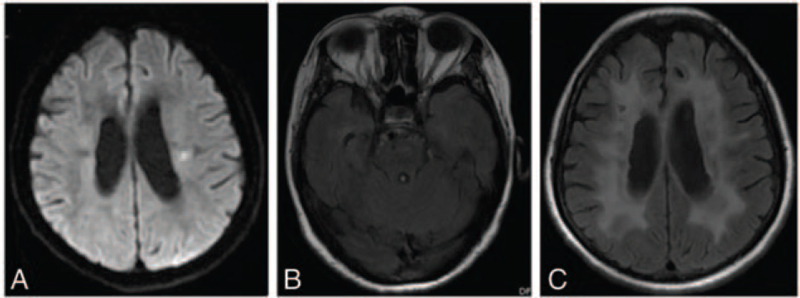
MRI images of the brain of the patient. (A) The diffusion-weighted image (DWI) shows acute lacunar subcortical infarction. (B) The axial T2-weighted image shows no white matter change in temporal lobes. (C) The fluid attenuated inversion recovery (FLAIR) shows extensive confluent white matter lesions at periventricular region.

**Figure 2:**
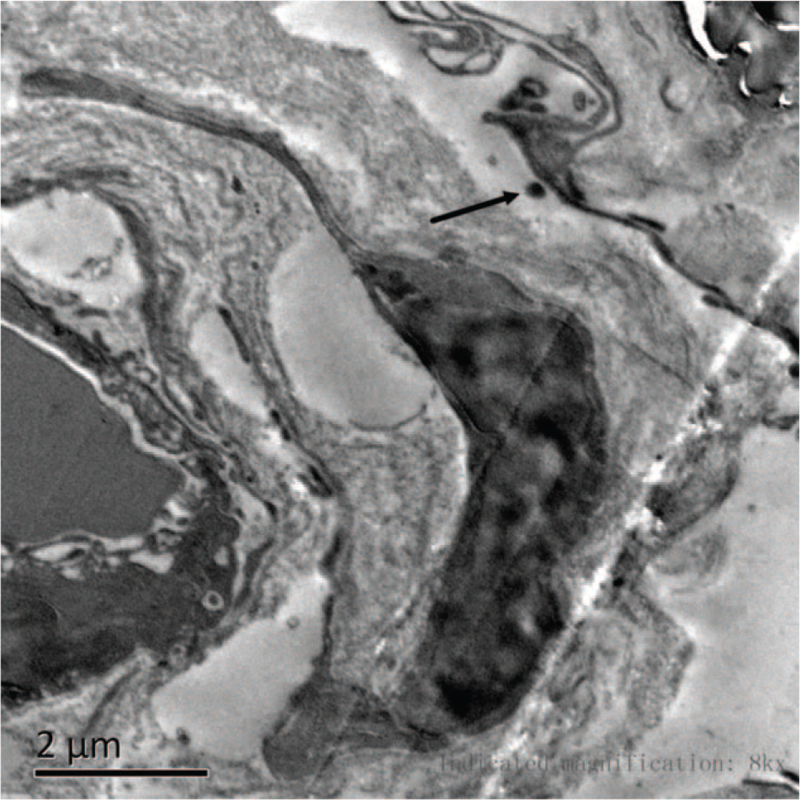
Pathological changes in the skin of the patient. Electron microscopy shows granular osmiophilic material (GOM), which is indicated by an arrow head, closes to the cell surface of smooth-muscle cells.

**Figure 3:**
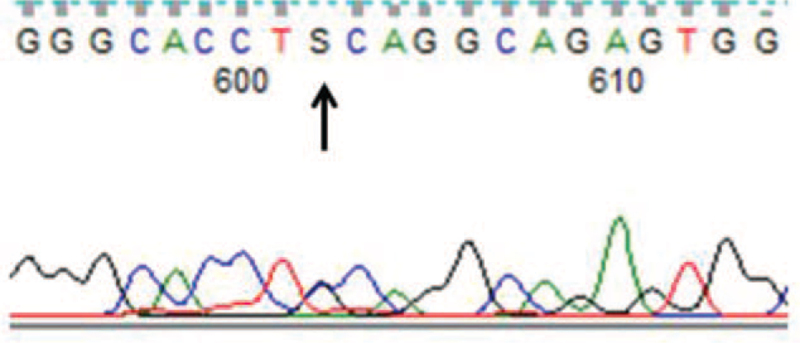
NOTCH3 gene analysis from DNA of the patient. The heterozygous c.635G>C[p.(Cys212Ser)] variant is indicated with an arrow head.

A 54-year-old woman presented with a history of 5 years of slurring of speech, recurrent weakness in both limbs and legs, and a 4 years of progressive memory decline. His Mini-Mental State Examination score was 18/30. She had no history of migraines. On neurological examination, she had forced laughter, the tendon reflexes in all of the limbs were very brisk, bilateral Babinski^,^s signs and Hoffman^,^s sign were positive, with a muscle strength of 4/5 in his upper limbs, and the power in the lower extremities was of grade 3/5 on the Medical Research Council scale. Diffusion-weighted imaging MRI and fluid-attenuated inversion recovery of the brain (Fig. 1) revealed recurrent acute lacunar subcortical infarctions and extensive white matter changes, without involvement of the temporal lobe. She had been diagnosed with ischemic cerebrovascular disease since her first episode and did not undergo regular antiplatelet therapy. However, she had no conventional vascular risk factors and a family history of minor stroke in her father and uncle at 46^,^ who then died at 53^,^s and 60^,^s respectively. The clinical manifestations, the presence of widespread leukoaraiosis and multiple lacunar subcortical infarctions, the absence of risk factors, and a positive family history made us suspect CADASIL. Subsequently, the patient underwent skin biopsy under local anesthesia. GOMs (Fig. 2) were detected close to the cell surface of smooth muscle cells in arteriolar vessels. The walls of several arteriolar vessels are thickened, loose, and layered. Genomic DNA was extracted from the peripheral blood. A mutation, c.635G>C[p.(Cys212Ser)], was detected in exon 4 (Fig. 3), leading to the substitution of cysteine with a serine in the EGF-like repeats. These findings allowed us to make a definitive diagnosis of CADASIL. Therefore, the patient was treated with antiplatelet therapy (aspirin 100 mg/day and clopidogrel 75 mg/day for 3 weeks, followed by aspirin 100 mg/day) and extremity rehabilitation training. However, the patient did not show any significant improvement in speech, extremity function, or memory. She has been followed up for 2 years with gradual worsening of her condition.

## 3 Discussion

Here, we report a novel pathogenic variant of NOTCH3 and its clinical, neuroimaging, and pathological presentations. The initial symptom of the disease was slurring of speech without a history of migraine, similar to the observations in a previous report.^[6]^ The patient suffered from recurrent lacunar infarction, but without vascular risk factors. The patient's father and uncle had similar symptoms. The MRI showed diffuse white matter hyperintensities, not including the temporal lobe lesions, indicating that temporal lobe lesions may not be characteristic of the disease, which is consistent with previous findings.^[^7^,^^8^^]^ Moreover, the patient who underwent skin biopsy showed GOM, which was highly consistent with molecular tests, implying that this type of mutation in CADASIL can be diagnosed by biopsy if genome sequencing is not available in some hospitals, unlike other types of NOTCH3 mutations in CADASIL, which are not sensitive to skin biopsy.^[^9^,^^10^^]^ The mutation c.635G>C[p.(Cys212Ser)] in exon 4 has never been recorded in HGMD (http://www.hgmd.cf.ac.uk/ac/search.php), leading to an odd number of one cysteine residue in the 34 EGFRs in the extracellular domain of the NOTCH3 protein; thus, it is a novel pathogenic variant of NOTCH3. From the description above, it is notable that the new NOTCH3 mutation reported here is correlated with an atypical presentation of CADASIL.

With advances in genetic testing and whole-exome/genome sequencing, more patients with CADASIL have been identified. Recently, Rutten et al^[11]^ found that patients with EGFrs 1–6 variants have an earlier stroke onset, higher brain lesion load, and lower survival rates than those with variants in EGFrs 7–34. Our patient with p. (Cys212Ser) mutation in exon 4 suffered recurrent acute lacunar subcortical infarction in the early stage, and her brain MRI showed serious lesions. Her condition progressed so rapidly that she was paralyzed completely within 5 years, which is consistent with Rutten's conclusion. In general, no treatment options for patients with CADASIL as a therapeutic target have been identified, and the disease is progressive and fatal.^[12]^

The relationship between the genotype and phenotype of NOTCH3 mutations is weak.^[4]^ It is difficult for neurologists to make a definitive diagnosis because of the highly variable phenotype. The patient was misdiagnosed several times. In conclusion, our study not only enriches the *NOTCH3* gene mutation spectrum, expanding our understanding of the genetic, clinical, and neuroimaging spectra of CADASIL but also, to some extent, could prevent misdiagnosis.

## Acknowledgments

The authors express their sincere gratitude to the patient for their understanding and participation in this study.

## Author contributions

**Conceptualization:** Jing Dang.

**Data curation:** Shengsuo Lei.

**Formal analysis:** Jing Dang, Mingwan Xia.

**Investigation:** Jihua Chen.

**Investigation:** Shengsuo Lei, Mingwan Xia.

**Methodology:** Jihua Chen.

**Resources:** Jing Dang.

**Writing – original draft:** Jing Dang.
